# Phylogeny and Biogeography of *Calanthe* Shed New Light on Alpine Origin and Radiation History of *Calanthe* Alliance

**DOI:** 10.1002/ece3.73301

**Published:** 2026-03-18

**Authors:** Jun‐Wen Zhai, Yanqiong Chen, Qiang Wang, Jiahao Zheng, Chen Chen, Pingting Guo, Hongyu Ji, Jianglin Miao, Muyang Li, Minghe Li, Shasha Wu, Siren Lan, Zhong‐Jian Liu

**Affiliations:** ^1^ Key Laboratory of National Forestry and Grassland Administration for Orchid Conservation and Utilization, College of Landscape Architecture and Art Fujian Agriculture and Forestry University Fuzhou China; ^2^ Institute of Oceanography, Department of Geography and Oceanography Minjiang University Fuzhou China

**Keywords:** biogeography, *Calanthe* alliance, hybridization, phylogenomic, plastome

## Abstract

The origin and radiation of plant lineages is one of the central topics in evolutionary biology, and the diversification of Orchidaceae is of more concern and still perplexing. In this study, we selected to resolve the origin and radiation of Orchidaceae by focusing on the *Calanthe* alliance. Using nrITS, plastome sequences, and single‐copy SNP data, we systematically reconstructed its phylogeny and spatiotemporal evolution. The monophyly of the six genera was confirmed; however, conflicts between the plastome and single‐copy SNPs were observed at both the species and generic levels. In addition, the Calanthe alliance originated in the late Oligocene, during a period of sharp climate cooling, while its three major clades were established in the warmest phase of the Cenozoic. The ancestral area of *Calanthe* was located in Southeast Asia, and the uplift of the Himalayas and the Tibetan Plateau in the Late Miocene drove the dispersal of *Calanthe* from tropics to subtropical and temperate areas. The diversification of the *Calanthe* alliance followed a two‐step pattern: an initial steady increase in lineages with adaptation to broader elevational ranges, and a second phase of relative stasis followed by recent speciation. Thus, we deemed that the montane area, as an essential distribution area of *Calanthe*, functioned as both “museum” and “cradle” in the diversification history of this genus. This work provides new insight into the classification and historical dynamics of complicated taxa in Orchidaceae.

## Graphical abstract

Using nrITS, plastome, and single‐copy SNP data, we resolved the phylogeny of the Calanthe alliance, which originated in the late Oligocene. Its diversification process followed a two‐step pattern: first the warmest Cenozoic interval and Himalayan uplift drove the formation of three major lineages, then sustained Miocene cooling shaped further diversification. Mountain environments have acted both as a “museum,” preserving ancient lineages, and as a “cradle,” facilitating the emergence of new species.

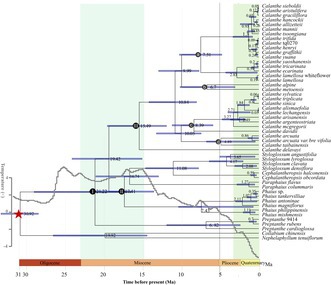

## 1 Introduction

Adaptive radiation of species plays an important role in facilitating the evolution and diversification of species, and biologists' interest in evolutionary radiations can be traced back to Darwin's “terrible mystery” (Soltis et al. 2019). In recent years, the mechanisms of adaptive radiation have been extensively studied in a number of plants, such as Lupinus (Drummond et al. 2012; Nevado et al. 2016, 2018), Lobelia (Givnish et al. 2009), Lepechinia (Drew and Sytsma 2013), and Rhododendron (Xia et al. 2021). Although these studies suggest that multiple herbaceous plant lineages may have undergone rapid adaptive radiation, existing research has largely focused on describing patterns and temporal dynamics of species diversification, with relatively limited attention to the underlying drivers of radiation. In particular, the interactive roles of intrinsic biological innovations and extrinsic factors such as geological processes, climatic changes, and ecological differentiation remain insufficiently explored.

Mountains harbor a disproportionately high level of biodiversity and are estimated to support nearly one‐third of terrestrial species diversity worldwide (Körner 2001), accounting for approximately half of the global biodiversity hotspots (Chape et al. 2008). The complex topography, pronounced elevational gradients, and strong geological and climatic dynamics of mountain systems provide abundant ecological opportunities that promote population isolation, local adaptation, and lineage diversification. Numerous studies have demonstrated that mountain uplift, climatic oscillations, and associated environmental heterogeneity play crucial roles in driving plant diversification and speciation (e.g., Ding et al. 2020; Wu et al. 2023). In particular, repeated climatic fluctuations and tectonic activities can generate spatiotemporally heterogeneous habitats, thereby shaping distinct evolutionary trajectories among plant lineages. Moreover, different life forms may respond to these environmental drivers in contrasting ways, leading to lineage‐specific patterns of diversification. Such differences highlight the importance of examining diversification processes within specific functional groups to better understand the mechanisms underlying mountain biodiversity, particularly in relation to climatic and geological dynamics. However, despite increasing attention to mountain biodiversity, the evolutionary histories and diversification processes of many herbaceous plant lineages in mountainous regions remain insufficiently explored.

The *Calanthe* alliance (Epidendroideae, Orchidaceae) comprises six genera—*Preptanthe*, *Phaius*, *Styloglossum*, *Cephalantheropsis*, *Paraphaius*, and *Calanthe*—with approximately 260 species, representing the largest lineage within Collabieae. Members of the alliance are predominantly terrestrial and only rarely epiphytic, and are morphologically characterized by plicate leaves, similar sepals and petals, and eight waxy pollinia arranged into two groups, features that readily distinguish them from other epidendroid orchids (Zhai et al. 2014). Traditionally, taxonomic classification and phylogenetic inference within the *Calanthe* alliance relied heavily on morphological traits, including a lip adnate to the column wing, spur morphology, and caducous bracts (van Breda 1827; Reichenbach 1853; Schlechter 1912; Chen et al. 2009). However, ambiguous morphological boundaries among species have long complicated generic and subgeneric delimitation within the alliance (Schlechter 1912; Pridgeon et al. 2005; Chen et al. 2009; Clayton and Cribb 2013).

Biogeographically, the *Calanthe* alliance is pantropically distributed, with major centers of diversity in Southeast Asia, the Malay Archipelago, and New Guinea (Clayton and Cribb 2013). In China, the alliance is particularly concentrated in the Hengduan Mountains (HDM), where a relatively high proportion of endemic species occurs. At the alliance level, *Calanthe* occupies a broad range of environments and elevations, extending from near sea level to over 3500 m, reflecting considerable ecological diversity across the clade. In contrast, at the species level, individual *Calanthe* species—especially endemic or narrowly distributed taxa—are often restricted to much narrower elevational ranges, suggesting limited elevational tolerance and/or pronounced habitat specialization (e.g., *C. delavayi*, 2700–3450 m; Lomolino 2001; Givnish et al. 2015). Such interspecific variation in elevational occupancy implies that environmental heterogeneity along altitudinal gradients may contribute to ecological differentiation and diversification within the alliance.

With the application of molecular data, phylogenetic relationships among genera within the *Calanthe* alliance have been substantially clarified (Chen et al. 2010; Yukawa 2013; Xiang et al. 2014; Zhai et al. 2014; Zhou et al. 2023; Ji et al. 2024), consistently demonstrating that *Calanthe* is polyphyletic. Nevertheless, although generic‐level relationships are now better resolved, interspecific relationships within genera remain weakly supported, and the circumscription of genera continues to be debated. Integrating molecular phylogenetics with evidence from biogeography, paleobotany, and climatology is therefore essential for reconstructing the evolutionary history of the *Calanthe* alliance (Zerega et al. 2005). Owing to its morphological diversity, wide geographic distribution, and contrasting patterns of ecological and elevational specialization among species, *Calanthe* provides an excellent model for investigating the evolutionary and historical biogeography of orchids.

To improve our understanding of the evolutionary and biogeography history of Calanthe alliance, we used a phylogenomic dataset from 49 Calanthe alliances to reconstruct the phylogeny. The results were used to (1) resolve the relationships of the infrageneric classification; (2) identify key nodes in the genus with underlying gene tree conflict and their causes; (3) reconstruct the spatio‐temporal evolution pattern of Calanthe alliance; and (4) infer how the function of the montane ecosystem plays roles in the speciation history of Calanthe alliance.

## 2 Materials and Methods

### 2.1 Sample Preparation

Fifty‐one terminals were used for genome skimming, comprising 32 species of *Calanthe*, four species of *Styloglossum*, three species of *Preptanthe*, two species of *Cephalantheropsis*, six species of *Phaius*, two species of *Paraphaius*, and the two outgroup taxa. Flowers from 18 species were collected for RNA transcriptome sequencing. These samples were selected as representative of six genera of *Calanthe* alliance, including 11 species of *Calanthe*, two species of *Styloglossum*, one species of *Preptanthe*, one species of *Cephalantheropsis*, two species of *Phaius*, and one species of *Paraphaius*. Transcriptome sequencing data were used to mine the single‐copy genes of *Calanthe* alliance. All taxa sampled with vouchers and GenBank accession numbers are listed in Table S1.

Total DNA was extracted from fresh leaf or silica‐dried leaf tissue with a modified version of the CTAB method (Doyle and Doyle 1987). Total genomic DNA was quantified using a Qubit 2.0 Fluorometer (Life Technologies, CA, USA). For genome skimming, the quantified DNA was used to construct sequencing libraries using the NEB Next Ultra DNA Library Prep Kit for Illumina (NEB, USA) following the manufacturer's recommendations. Libraries were pooled and run on an Illumina HiSeq 4000 platform, and 150‐bp paired‐end reads were generated. The raw data of each sample were filtered by removing adapters, as well as short and low‐quality sequences, using Trimomatic 0.3 (Bolger et al. 2014) with the default parameters.

Total RNA was extracted from the flower tissue samples using the Spectrum Plant Total TNA Kit (Sigma; Saint Louis, MO, USA). Transcriptome library construction and sequencing were performed by BioMarker Technologies (Beijing, China).

### 2.2 NGS Sequence Assembly, Single‐Copy Gene Identification and SNP Calling

For genome skimming, paired‐end reads were assembled using GetOrganelle pipeline v1.7.1 (Jin et al. 2020) with a *k‐mer* range of 21, 45, 65, 85, and 105 for plastomes. The published plastome *Calanthe triplicata* (KF753635) was used as a reference for the plastome assembly. Geneious prime v2020.0.5 was used (Kearse et al. 2012) to manually align the results and annotate them with *C. triplicata* as a reference (KF753635) to obtain high‐quality complete plastomes of *Calanthe* alliance. The graphical maps were drawn using OrganellarGenomeDRAW (Lohse et al. 2007).

The nrITS (18S‐ITS1‐5.8S‐ITS2‐28S) sequence was assembled using GetOrganelle pipeline v1.7.1 (Jin et al. 2020) with a *k‐mer* range of 35, 85, and 115, with the initialized database “embplant_nr” as a reference. Geneious prime v2020.0.5 (Kearse et al. 2012) was applied to annotate the ribosomal RNA genes and their boundaries with ITS regions using *Calanthe bicolor* nrITS sequence (MN221391) as a reference.

For transcriptome sequencing, the raw reads were filtered to obtain high‐quality clean reads by removing the adapter sequences or low‐quality reads, as well as plastid and mitochondrial sequences. The clean reads were deposited in the NCBI Short Read Archive (SRA) under the accession number PRJNA766248. The clean reads were de novo assembled using Trinity (Grabherr et al. 2011), with min_kmer_cov set to 2 by default and all other parameters set to the default. Gene function was annotated based on the following databases: NR (NCBI nonredundant protein sequences); Pfam (Protein family); KOG/COG/eggNOG (Clusters of Orthologous Groups of proteins); Swiss‐Prot (a manually annotated and reviewed protein sequence database); KEGG (Kyoto Encyclopedia of Genes and Genomes); and GO (Gene Ontology). The annotated sequences were aligned with all‐to‐all blast by OrthoMCL (Li et al. 2003) with an e‐value cutoff of 10‐5 for gene family clustering. 1260 single‐copy genes were identified, and this dataset was used as a reference for SNP calling.

Compared with using reconstructed nuclear gene sequences from low‐coverage skimming data, the SNP dataset provides denser, more widely distributed nuclear information and is less sensitive to assembly artifacts. The BWA v0.7.17 (Li and Durbin 2009) was used to map the genome skimming paired‐end reads for all taxa to the single‐copy gene dataset. The aligned reads were filtered to remove PCR duplicates and sorted according to mapping coordinates with the PICARD package v 2.17.5 (http://broadinstitute.github.io/picard/). The circumindel regions were realigned using the Genome Analysis Toolkit (GATK; McKenna et al. 2010). The resulting alignment files were subjected to genotyping using GATK UnifiedGenotype. A total of 167,374 SNPs were identified.

### 2.3 Phylogenetic Analysis

For the phylogenetic analysis, the following matrices were investigated: (1) plastome data, in which only one copy of the IR was included (CP), (2) nrITS dataset (nrITS), and (3) single‐copy SNP data (SNP). All datasets included 51 accessions representing 50 species.

Bayesian inference (BI), maximum parsimony (MP), and maximum likelihood (ML) methods were used for phylogenetic reconstruction. The BI, MP, and ML analyses were run on the CIPRES Science Gateway (RAxML‐HPC2 on XSEDE 8.2.12; Miller et al. 2010), MrBayes on XSEDE 3.2.7a, PAUP on XSEDE 4.a168, and RAxML‐HPC2 on XSEDE 8.2.12. For the BI analysis, the GTR + T + Γ model was specified for all datasets (Ronquist et al. 2012). The Markov chain Monte Carlo (MCMC) algorithm was run for 10,000,000 generations, with one tree sampled every 100 generations. The first 25% of trees were discarded as burn‐in to ensure that the chains reached stationarity. The 50% majority‐rule consensus tree and posterior probabilities (PP) were calculated from these trees sampled after generation 10,000,000. MP analyses were performed using 1000 tree‐bisection‐reconnection (TBR) searches with MAXTREES set to increase without limit (Swofford 2003). All characters were equally weighted and unordered, and a heuristic search with 1000 random addition sequence replicates and TBR branch swapping was performed. For the ML analysis, the ASC_GTRCAT model was specified for all dataset to account for the absence of constant characters during ML tree estimation, and node support was similarly evaluated using 1000 bootstrap replicates (Stamatakis 2014).

### 2.4 Divergence Time Estimation

We inferred the divergence times of *Calanthe* alliance based on the plastome dataset. There are only three fossils in Orchidacea (*Dendrobium winikaphyllum*, *Earina fouldenensis*, and *Meliorchis caribea*; Conran et al. 2009; Ramírez et al. 2007), and none of them are related to *Calanthe* alliance. Secondary calibrations were applied to reduce the calibration bias from distantly related taxa (Ehl et al. 2019; Lai et al. 2021; Xiang et al. 2016). Using two loci (*matK* and *rbcL*), Xiang et al. (2016) constructed major orchid lineages and generated a fossil‐calibrated tree. The crown age of Tribe Collabieae estimated by Xiang et al. (2016) (offset = 30.49, mean = 0.5, sigma = 1) was used to generate rate‐corrected and time‐calibrated phylogenetic trees.

Based on the total matrix dataset, the Yule speciation process (Gernhard 2008) was presumed and BEAST v.1.10.4 (Drummond and Rambaut 2007) was used on the CIPRES gateway (Miller et al. 2010), along with the Bayesian MCMC method under an uncorrelated lognormal relaxation clock. BEAUti v.1.10.4 was used to create the BEAST input file. Separate data partitions were defined in different sequences, and model parameters were unlinked between partitions. Two independent MCMC analyses of 50,000,000 steps each with the first 25% of steps discarded to calculate the posterior distributions of parameters. Convergence on the same distribution and mixing were assessed to ensure an effective sample size (ESS) using Tracer v1.7.2 (Drummond and Rambaut 2007). The samples from the posteriors were summarized using the maximum clade credibility (MCC) tree option in TreeAnnotator v.1.10.4 (distributed with BEAST). The final tree, supports and ages were visualized using FigTree 1.4.2.

### 2.5 Ancestral Area and Elevation Reconstructions

Two methods were used in ancestral area reconstructions: the Bayesian statistic parsimony‐based method (S‐DIVA; Yu et al. 2010) and maximum likelihood approach under the dispersal‐extinction‐cladogenesis (DEC) model (Ree and Smith 2008). Both analyses were implemented in RASP v.3.1 (Yu et al. 2015).

Bayesian molecular dating trees obtained from BEAST with 1000 tree discards as burn‐in were treated as an input for the S‐DIVA and DEC analyses. The condensed tree obtained from the MCC tree produced in the BEAST analysis. Based on the distribution range of sampled species, five biogeographic areas were recognized: (A) Eastern Asia (including China, Japanese Islands, and Korea Peninsula); (B) India and Indochina; (C) Malaysia and Australia; (D) Africa; (E) Central America. The altitude distribution range data of *Calanthe* alliance were collected according to Chen et al. (2009), Clayton and Cribb (2013) and our field observations (see Table S2).

In addition to ancestral area reconstruction, ancestral elevation reconstruction was conducted to investigate the evolutionary history of elevational preference within the *Calanthe* alliance. Elevation was treated as a continuous trait, using the midpoint of the recorded elevational range for each species. Elevational data were compiled from the Flora of China (Chen et al. 2009), Genera Orchidacearum (Clayton and Cribb 2013), and our own field observations (Table S2).

Ancestral elevation reconstruction was performed under a maximum‐likelihood framework using the time‐calibrated phylogeny. Alternative models of continuous trait evolution were compared, and the λ (lambda) model was selected as the best‐fitting model based on model likelihood scores. The reconstructed ancestral elevation values were mapped onto the phylogeny to visualize elevational evolution across major lineages of the *Calanthe* alliance.

### 2.6 Diversification Rate Heterogeneity Analyze

We used BAMM v 2.2.1 (Bayesian Analysis of Macroevolutionary Mixtures) to infer the diversification rate heterogeneity (Rabosky 2014). The analysis use run jump MCMC approach with 10,000,000 iterations. R package “coda” (Plummer et al. 2006) was implemented to tested the MCMC output to ensure convergence (effective sample sizes (ESS) for likelihood and number of shifts > 1000), with a burn‐in of 10%. The results were used to calculate diversification rates with “BAMMTools” 2.0.2 in R package (Rabosky et al. 2014). GetEventData and plot.bammdata functions was used for generating phylorate plot. LTT plots derived from the maximum clade credibility tree and the temporal dynamics of diversification in the *Calanthe* was measured by using the R package “ape” (Paradis et al. 2004). BEAST analysis 100 random trees were used in plots.

### 2.7 Ancestral Area and Elevation State Reconstruction

Four important diagnostic morphological characters are used to define infrageneric taxa of *Calanthe* alliance: (1) lip adnate to column wings, (2) spur, (3) lip lobe, and (4) lip appendage. The ancestral state and evolution of these characters were assessed by tracing the character onto plastome Bayesian trees using the trace character over trees option and the maximum likelihood approach with the Markov k‐state one‐parameter (Mk1) model implemented in Mesquite v3.70 (Maddison 2009). Morphological information was obtained from the description in the Flora of China (Chen et al. 2009) and Clayton and Cribb (2013; see details in Table S3).

## 3 Results

### 3.1 Phylogenetic Analysis

**FIGURE 1:**
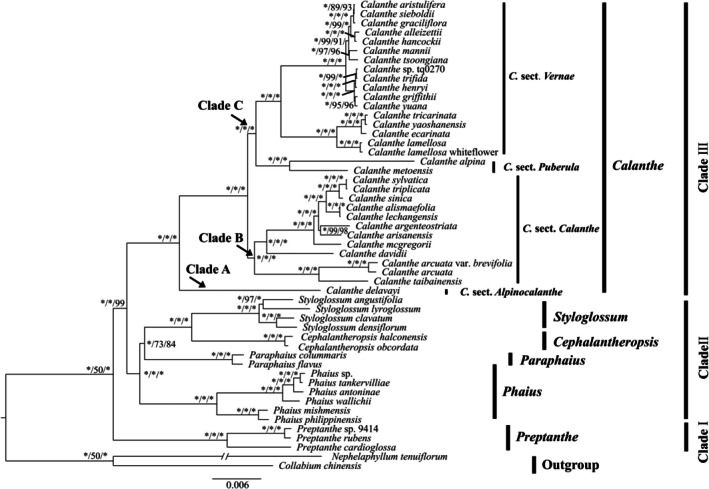
Bayesian inference based on the plastome sequences. Numbers near the nodes are Bayesian posterior probabilities and bootstrap percentages (PP left, BS_ML_ middle, and BS_MP_ right). An asterisk (*) indicates that the node has 1.0 posterior probability or 100% bootstrap.

Fifty‐one species were sampled, and three datasets (plastome, nrITS, and single‐copy SNPs) were generated to capture complementary evolutionary signals derived from maternally inherited plastomes, biparentally inherited nuclear ribosomal DNA, and genome‐wide nuclear markers, respectively (Figure 1; Figures S1 and S2). The relationships among major clades were determined and strongly supported across all analyses, and the interspecies phylogeny was of high resolution in two genome‐wide datasets. Considering that plastome phylogenies provide a single, low‐recombining locus that is particularly suitable for divergence time estimation and historical biogeographic reconstruction, reducing analytical complexity and potential violations of model assumptions. Therefore, the plastome‐based ML tree constructed from the concatenated matrix of 51 plastoms (CP) was used as the primary framework for further diversification and biogeographic analyses.

All six genera were resolved as monophyletic and the *Calanthe* alliance was divided into three strongly supported clades (Figure 1). Clade I has only one genus, namely, *Preptanthe*, which was the earliest‐diverging lineage (PP = 1.00, BS = 100). Clade II contained 14 species of four genera, namely, *Phaius*, *Paraphaius*, *Cephalantheropsis*, and *Styglossum. Phaius* was shown to be sister to the other three genera (PP = 1.00, BS = 100). *Styloglossum* and *Cephalantheropsis* formed a clade sister to *Paraphaius*. The last clade included the remaining species of *Calanthe* and was subdivided into three subclades, A, B, and C (PP = 1.00, BS = 100). The first subclade A consists of only one species, *C. delavayi*. The second divergent clade contained 12 species of sect. *Calanthe*. The third clade was composed of two sister groups, sect. *Puberula* and sect. *Vernae*.

**FIGURE 2:**
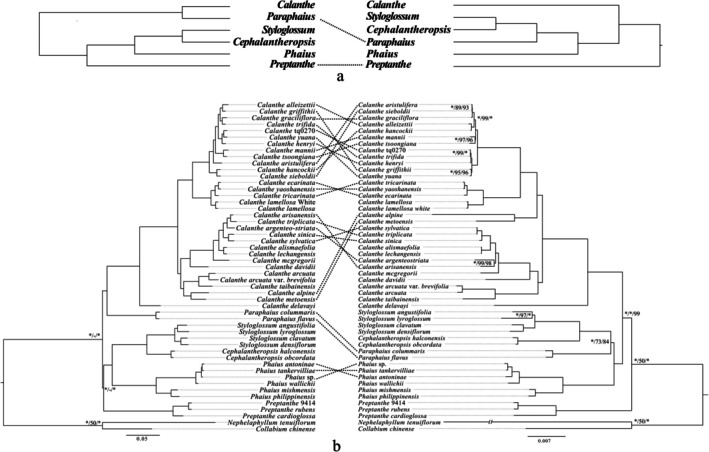
Comparison between phylogenetic topologies inferred from single‐copy nuclear SNP data (left) and plastome data (right) for the Calanthe alliance. (A) Simplified topological comparison at the generic level, highlighting major congruent and incongruent relationships among genera inferred from the two datasets. (B) Detailed species‐level phylogenetic comparison, showing relationships within and among genera of the Calanthe alliance based on the same datasets. Numbers near the nodes indicate Bayesian posterior probabilities and bootstrap support values (PP on the left, BS_ML in the middle, and BS_MP on the right). An asterisk (*) denotes nodes with maximum support (PP = 1.0 and/or bootstrap = 100%). For clarity, Bayesian posterior probabilities and bootstrap values for nodes with low support or in densely sampled clades are omitted.

Incongruence was significant among the topologies obtained from the plastome, nrITS, and single‐copy SNP data (Figure 2). The phylogeny inferred from nrITS was unstable (the topology obtained by the three analysis methods was incongruent, and the resolution between nodes was low), and we only discussed conflict between the other two datasets. The conflict appeared frequently in sect. *Vernae*, as 15 species (out of 17 species in this study) had different topologies in two datasets. Only three incongruences were found in sect. *Calanthe*. The positions of sect. *Puberula* (*C. alpine* and *C. metoensis*), *Paraphaius* (*Para. flavus* and *Para. colummaris*) and *Preptanhe* (*Pre*. sp. 9414, *Pre. rubens*, and *Pre. cardioglosa*) were significantly different in plastome and SNP phylogenetic results. The last genus with conflicting topologies is *Phaius*, and three species showed differences. Only two genera had consistent topologies in two datasets, namely, *Styloglossum* and *Cephalantheropsis*.

### 3.2 Molecular Dating

**FIGURE 3:**
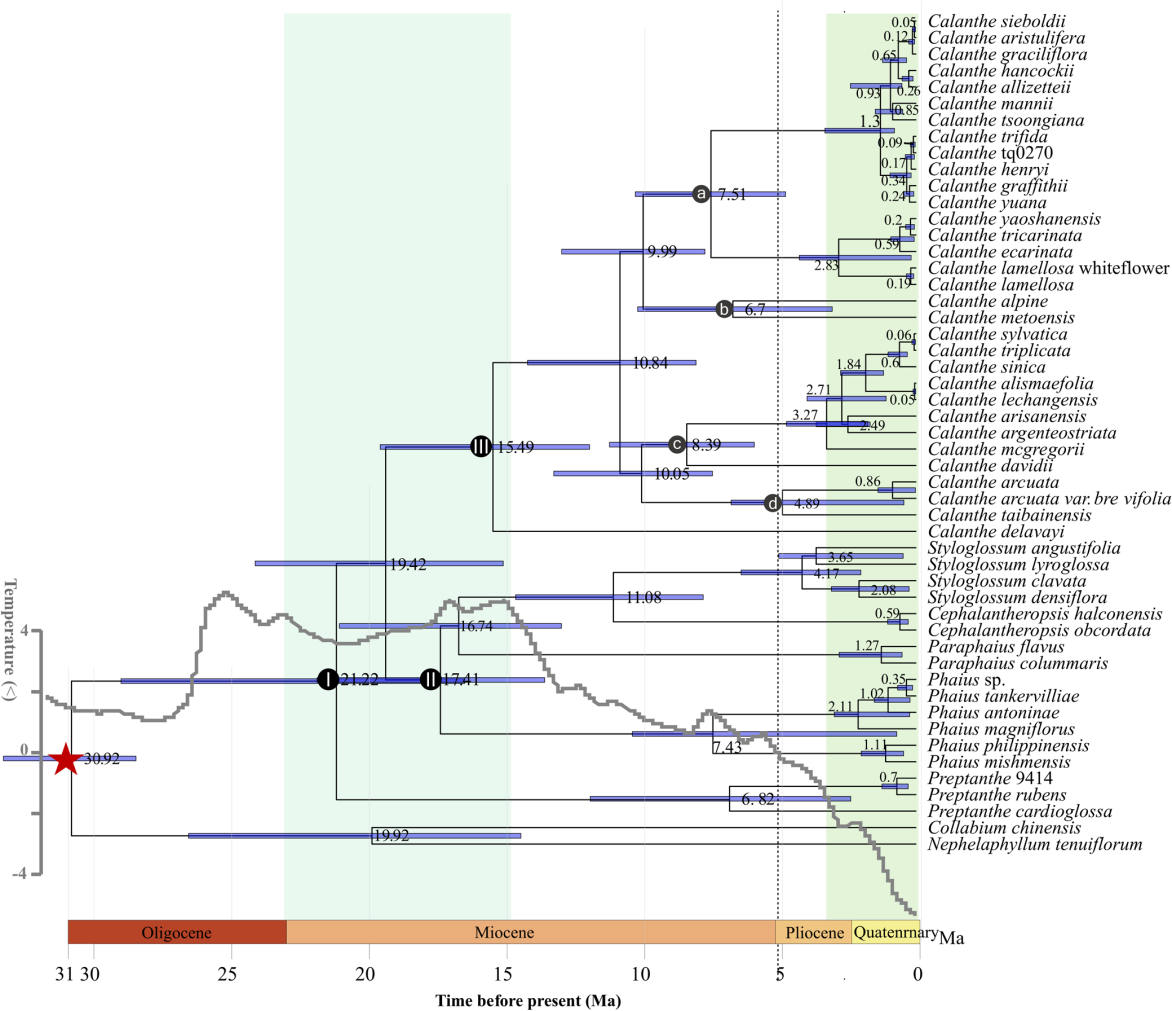
Beast maximum clade credibility tree inferred from the plastome sequences. The divergence times of each clade are displayed near each node. Blue bars represent the 95% highest posterior density for the node ages. The gray line represents the temperature curve since the Miocene. The gray line represents the global mean temperature curve since the Miocene, reconstructed from published paleoclimatic data, and is included to provide a temporal climatic framework for comparing lineage divergence events with major climatic transitions. The two vertical green shaded areas indicate major climatic transition periods. The left green belt corresponds to the Mid‐Miocene Climatic Optimum (MMCO), whereas the right green belt represents the period of post‐Miocene global cooling from the late Miocene to the Pliocene–Quaternary. Dark gray circles indicate sectional classifications within *Calanthe*. Letters a, b, c, and d correspond to sect. *Vernae*, sect. *Puberula*, sect. *Calanthe*, and sect. *Alpinocalanthe*, respectively.

The divergence times were estimated based on plastome sequences. According to our estimates of divergence time (Figure 3, Table S4), the stem node of Calanthe alliance originated in the late Oligocene (30.92 Ma, 95% HPD = 28.56–33.41). Preptanthe was first divergent at 21.22 Ma. Clade II was estimated to occur in the late Miocene (17.41 Ma, 95% HPD = 13.59–21.93 Ma). The last evolved Calanthe was split with other genera in the mid‐Miocene (15.49 Ma, 95% HPD = 11.95–19.62 Ma). And it was found that most species of sect. Vernae have diverged quite recently.

### 3.3 Ancestral Area and Elevation Reconstructions

**FIGURE 4:**
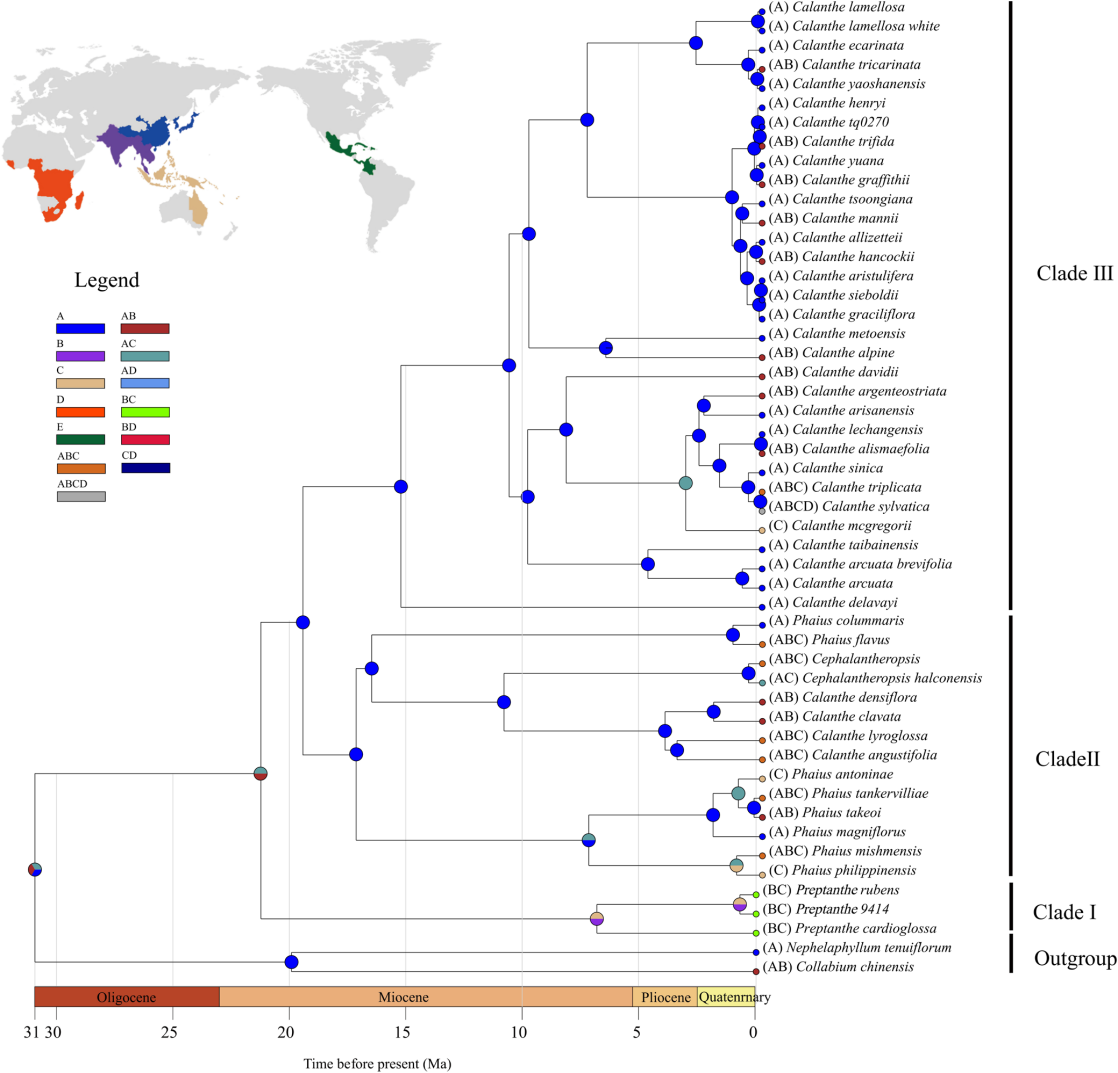
Ancestral area reconstruction of *Calanthe* alliance as inferred with Statistical Dispersal‐Vicariance Analysis (S‐DIVA) in rasp, using 1000 random Bayesian trees from the beast analysis. Nodal probabilities of ancestral areas are depicted on the Bayesian tree of Figure 2. Single‐colored pie diagrams indicate an ancestor confined to a single geographic area; multi‐colored pie diagrams represent the probabilities of different areas at each node. Arrows and circles represent dispersal and vicariance events, respectively. Biogeographical region abbreviations: (A) Eastern Asia; (B) India and Indochina; (C) Melasia and Australia; (D) Africa; (E) Central America. The gray line represents the temperature curve since the Miocene.

Southeast Asia seems as the possible ancestral area for the *Calanthe* alliance both in S‐DIVA and DEC analyses and they then diverged into mainland Asia, Australasia, Central America and Africa. The result of the S‐DIVA analysis of optimal ancestral range reconstructions was used and plotted in the time framework phylogeny tree (Figure 4). The most recent common ancestor (MRCA) of all the three main clades had been shown to originate in mainland Asia.

In addition to geographic reconstruction, we performed an ancestral state reconstruction of elevational preference to explicitly evaluate the evolutionary history of elevation within the *Calanthe* alliance. Elevation was treated as a continuous trait and reconstructed under a maximum‐likelihood framework. The reconstructed elevational states suggest that the MRCA of the *Calanthe* alliance likely occupied mid‐ to high‐elevation habitats, consistent with montane environments in tropical Southeast Asia. Subsequent diversification involved both upward and downward shifts in elevational range across different lineages. In particular, several derived clades expanded into lower‐elevation tropical habitats, whereas others diversified further into higher‐elevation montane and subalpine zones, especially in mainland Asia.

### 3.4 Diversification Rate Heterogeneity Analyze

**FIGURE 5:**
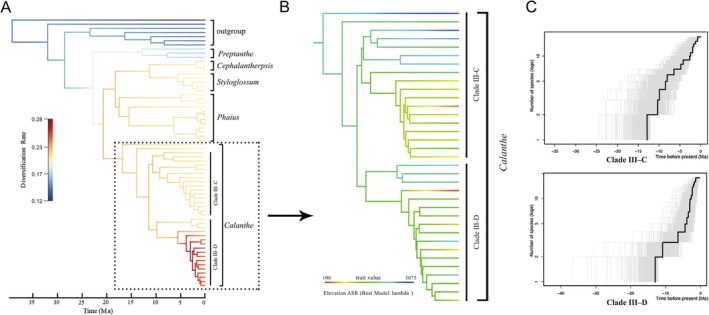
(A) A phylorate plot showing the heterogeneity of diversification rates in Calanthe alliance estimated from BAMM. (B) the elevation reconstruction of *Calanthe*. (C) log lineage‐through‐time plot for the Clade III–C and Clade III–D, Gray lines represent the LTT plots for 1000 trees randomly selected from the beast analysis. The black line shows the maximum clade credibility tree.

The BAMM analysis revealed a highly heterogenous diversification rate among different clades (Figure 5A). In the Pliocene and Quaternary Clade IV–D showed highest diversification rate. The elevation ranges of each *Calanthe* species were marked in the phylogeny tree (Figure 5B). The elevation range changed (from high to lower elevation) with the evolution process of *Calanthe*, especially Clade IV–D (Figure 5B). LTT analysis indicated that *Calanthe* did not exhibit a constant speciation rate, and an increase in the diversification rate of Clade IV was due to recent radiation; in particular in subclades C and D, in a different diversification path (Figure 5C).

### 3.5 Morphological Character Evolution

**FIGURE 6:**
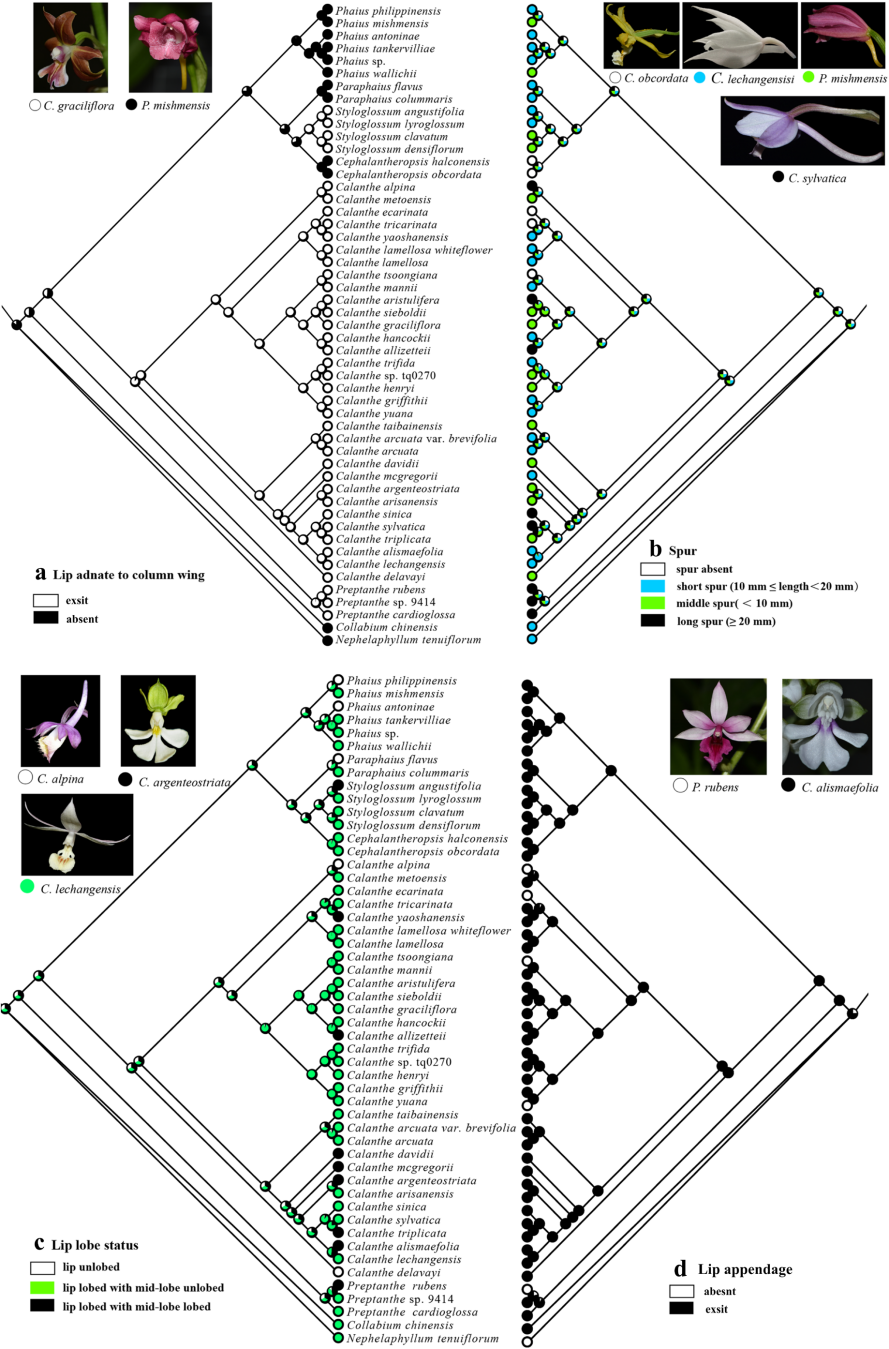
Ancestral state reconstructions of lip adnate to column base (A), spur length (B), lip lobe status (C), and lip appendage (D) in the *Calanthe* alliance. The likelihood of occurrence of each state at each node is indicated by colored wedges of different sizes. The images on the upper left and upper right show the characteristics of representative taxa of the four morphologies. Photographs by the co‐authors.

The reconstruction of ancestral morphological characters indicated that the evolution patterns of *Calanthe* alliance are complicated. The lip unadnate to the column base, spur, lip in lobed status and lip with appendage are the ancestral states of *Calanthe* alliance (Figure 6). Lip unadnate to the column base was lost at least three times in *Preptanthe*, *Calanthe*, and *Styloglossum* (Figure 6A). The spur's existence was lost three times in *Calanthe* and *Cephalantheropsis*. The spur length exhibited rampant diversity and complex evolutionary patterns (Figure 6B). Our data suggested that its ancestral state was equivocal. Lip lobed status was lost at least five times in Clades II and III (Figure 6C). Lip without appendage evolved at least five times in *Preptanthe* and *Calanthe* only (Figure 6D).

## 4 Discussion

### 4.1 Phylogenetic Relationships of *Calanthe* Alliance

This study presented the first genome‐level phylogenetic study of *Calanthe* alliance using plastome, total nrITS and single‐copy SNP data. The datasets indicated that *Calanthe* alliance are monophyletic and that all six genera are recognized as monophyletic. This result reinforced the previous conclusions from studies using few DNA markers (Xiang et al. 2014; Yukawa and Cribb 2014; Zhai et al. 2014). Consistent with a previous study (Xiang et al. 2014; Yukawa 2013; Zhai et al. 2014), the plastome and SNP datasets supported *Preptanthe* as a basal clade of *Calanthe* alliance. The morphological characteristics of *Preptanthe* were also significantly different from those of other genera, including deciduous leaves and large flesh pseudobulbs. *Cephalantheropsis* and *Styloglossum* was closely related to *Phaius* or *Paraphaius* (Figure 2A), which is inconsistent with previous results (Xiang et al. 2014; Zhai et al. 2014). In Zhai et al. (2014), Xiang et al. (2014) and Zhou et al. (2023), *Phaius* was the second divergent genus, but weak support was observed. Yukawa (2013) split *Phaius* into three independent groups but without resolution. In our analyses, the two genome‐wide datasets present *Phaius* sister to the clade comprised of *Styloglossum* and *Cephalantheropsis. Paraphaius* obtained three different positions using the three datasets in this study, but only the SNP dataset shaped the robust phylogenetic result (in a sister relationship with *Calanthe* clade).

*Calanthe* is the most diverse and species‐rich group of *Calanthe* alliance (Clayton and Cribb 2013). Two genome‐wide trees established three main clades (A, B and C; Figure 1 and Figure S2). *C. delavayi* formed clade A, which was named sect. *Alpinocalanthe* (Zhai et al. 2014), and our results supported this treatment in both datasets. In clade C, *C. alpina* and *C. metoensis* formed a clade, which is isolated from sect. *Vernae*; it is close to sect. *Vernae* in the plastome genetics; while in SNPs dataset, it was nested in sect. *Calanthe* (sister to the clade composed of *C. arcuata* , *C. arcuata* var. *brevifolia*, and *C. taibainensis*); two datasets both obtained a robust relationship (Figure 2B). Morphologically, *C. alpina* and *C. metoensis* are similar to the *arcuata‐taibainensis* clade; they are all identified by their small individual, elongated petals, and fimbriate or undulate dentate lip apical margins, and are distributed in montane to high‐altitude habitats, with recorded elevations reaching up to 3300 m. Within sect. *Vernae*, almost all the species in this study (except for *C. lamellosa* ) obtained a conflicting topology between the two genome‐wide datasets (Figure 2B). This extensive discordance is likely attributable to a combination of rapid recent radiation and the resulting extremely short internodes, which limit the accumulation of informative nuclear variants and enhance the effects of incomplete lineage sorting. In addition, the predominantly montane distributions, recent divergence times, and overlapping geographic ranges of many *Vernae* species suggest that historical or ongoing hybridization and plastid capture may have further contributed to the observed plastome–nuclear incongruence.

Furthermore, the tree constructed by plastome, nrITS, and SNP data showed a short branch in all species (Figures 1 and 2; Figures S1 and S2). This phenomenon was also found in previous molecular phylogeny analyses (Zhai et al. 2014). Species have likely experienced rapid speciation, with little time for informative mutations to accumulate (Glor 2010), and the phylogenetic resolution is very low in the datasets with fewer informative loci, for example, nrITS. Arbitrating between fast speciation would be particularly challenging given the very short branch lengths. This suggests a complex evolutionary history for sect. *Vernae*, but different gene trees could provide interesting insights into recent evolution and possible hybridization events.

### 4.2 The Evolutionary History of *Calanthe* Alliance

Hybridization has long been recognized as an important contributor to the assembly of plant diversity over time (Abbott et al. 2013). In the Orchidaceae, the most diverse angiosperm in the world, numerous hybridization events have been proposed in *Epidendrum* (Marques et al. 2014; Vega et al. 2013), Dactylorhiza (Brandrud et al. 2020; De Hert et al. 2012), and *Spiranthes* (Pace et al. 2019; Surveswaran et al. 2018). In *Calanthe*, some species have been reported to be involved in hybridization events, for exampl, *C. discolor*, *C. izu‐insularis* (Nakahama et al. 2019), and the natural hybrid *Calanthe* × *hsinchuensis* (Lee 2013).

Our results suggest that both hybridization and incomplete lineage sorting have shaped the evolutionary history of the *Calanthe* alliance, but their relative importance varies among clades. Extensive plastome–nuclear discordance concentrated within sect. *Vernae*, together with its recent divergence and sympatric distributions, is consistent with a scenario involving both incomplete lineage sorting and possible hybridization. Although introgression has been reported in a limited number of Vernae species (Liu et al. 2020), whether recurrent hybridization and chloroplast capture are widespread across the entire section remains uncertain. In contrast, limited discordance in sect. *Calanthe* and the pervasive short internodes in Clade III–D are more consistent with incomplete lineage sorting following rapid radiation. At deeper nodes, such as the placement of *Paraphaius*, ancient hybridization or plastid capture may have contributed to the observed topological instability. These findings highlight a complex, reticulate evolutionary history in Calanthe, particularly in montane regions where rapid diversification and secondary contact are likely to have been frequent (Rieseberg and Soltis 1991).

At deeper nodes, such as the placement of Paraphaius, the observed topological instability may reflect a combination of ancient rapid divergence, limited phylogenetic signal, and heterogeneity between plastid and nuclear genomes. Although ancient hybridization or plastid capture cannot be excluded, alternative explanations should also be considered given the lack of direct evidence.

Dynamic geological historical events and climatic fluctuations were inextricably interwoven with evolutionary processes (De Bruyn et al. 2014; Guo et al. 2015; Xing and Ree 2017). Our dating analyses suggested that *Calanthe* alliance experienced two major diversity events. The first diversity occurred in the early–middle Miocene, when all six genera diverged (Figure 3). The establishment of the three major clades of the Calanthe alliance during the early–middle Miocene (~23–15 Ma) corresponds to the Miocene Climatic Optimum and the second major uplift phase of the Himalayas and Tibetan Plateau. The interaction between warm climates and rapid mountain building likely expanded montane forest habitats and created novel elevational niches. These newly available habitats would have facilitated upslope dispersal and ecological divergence from lowland tropical ancestors, promoting rapid lineage splitting within a relatively short time frame (Herold et al. 2011; Reuter et al. 2013), providing suitable conditions for evolution. Early diversification accompanied by ancient hybridization events may explain the incongruence of the six genera in different phylogenetic trees. The second diversity happened within 5 Ma. Most of the conflicting phylogenetic results between plastomes and SNPs were within this time frame. The turbulent climatic shift of the Quaternary had a dramatic effect on species evolution. Species had to survive the coldest periods in refugials and recurrently colonized during interglacial periods (Kerdelhué et al. 2009). This recent diversity may be explained by hybridization or Incomplete Lineage Sorting, which could occur frequently between or within species with overlapping flowering periods and sympatric distributions. There was a complicated divergent history within *Calanthe*, especially in sect. *Vernae*. This last divergent section has abundant morphological features (Figure 6), e.g., various appendage types of lip disks: calli, lamellates or ridges, spurs absent or of different lengths. In this clade, interspecific hybridization has been inferred, e.g., introgression among *C. yaoshanensis* , *C. tricarinata* , and *C. lamellosa* , and three sympatric species have been detected (Liu et al. 2020). Hybridization might be frequent during evolutionary bursts of speciation; ancient and recent diversifications (and radiations) are especially prone to the phylogenetic conflict explained here.

### 4.3 Tropical Origin and Two‐Step Diversification History of *Calanthe* Alliance

Ancestral state reconstruction indicated that the *Calanthe* alliance most likely originated in tropical regions, as the earliest diverging lineages are restricted to tropical Southeast Asia and adjacent areas. Subsequent diversification involved multiple dispersal events, leading to range expansion into subtropical and temperate East Asia and the evolution of six distinct genera. The establishment of the three major clades (Clade I–III) during the early–middle Miocene (~23–15 Ma) coincided with a period of significant paleoclimatic and paleogeographic reorganization in Asia. This interval encompassed the Miocene Climatic Optimum, the onset and intensification of the East Asian monsoon system (Sun and Wang 2005), and the major uplift phases of the Himalayas and Tibetan Plateau. The interplay of these factors likely transformed regional climatic patterns, enhancing seasonality and environmental heterogeneity across Southeast and East Asia. Such shifting environmental landscapes may have provided ecological opportunities that promoted the initial divergence and adaptive evolution within the alliance. For instance, the earliest‐diverging lineage (Clade I, *Preptanthe*), which is restricted to regions with pronounced seasonal climates, exhibits deciduous leaves and fleshy pseudobulbs—traits often associated with adaptation to seasonal drought. While the exact selective pressures driving the evolution of deciduousness in *Preptanthe* require further investigation, its divergence during this dynamic period suggests a potential link between broader climatic shifts and the emergence of key ecological adaptations within different lineages of the alliance. Clade II has been shown to diverge in mainland Asia and presents a stem age at 17.41 Ma (Table S4) and limited geographic distribution in Southeast Asia. Clade III split up from Clade II at 15.49 Ma and then diverged into two separated clades immediately. The most recent genus *Calanthe* originated in the montane region of Asia and then diversify.

Two‐step diversification history was detected in *Calanthe* alliance. The genus emerged from Southeast Asia eastward and got distributed to mainland Asia and far to the Japanese archipelago and then into America. It then dispersed southward to Australia ( *C. triplicata* ) and westward to Africa, and thus, formed the disjunctive distribution we observe today, consistent with the results of Ji et al. (2024). The first step happened between 23–17 Ma, and the genus rapidly diversified into all the three major clades (Clade I–III) (Figure 4). This stage was consistent with the second phase of the upward movement of the Himalayas and the Tibetan Plateau that happened in later Oligocene‐early Miocene (Tapponnier 2001). Additionally, the climate was very warm and the temperature was very high in this period during the Cenozoic era (Zachos et al. 2001) that may promote the origin and early diversification of the three major clades. A similar scenario has been detected in *Lilium* (Gao et al. 2013) and *Cyananthus* (Zhou et al. 2013). Later, the second step was a more recent diversification in the Miocene, during the time a significant climate cooling period may have led to a habitat reduction in the distribution of both the tropical forests and the coniferous forests in the continent. Furthermore, continuous cooling temperature ushered in the glacial period, which fragmented the habitats of many organisms, while simultaneously forming shelters (Gong et al. 2016; Wurster et al. 2010; Zhou et al. 2017), leading to vicariance and the opportunity of secondary contact, which may be the possible reasons for the diversification of major extant taxon.

### 4.4 Function of the Montane Ecosystem in Speciation History of the *Calanthe*

Variable mountain ecology along elevation gradients has been always seen as a “diversity pump,” which may have led to the origin of a large portion of the world's biodiversity (Segar et al. 2017). Species diversity in mountainous areas could be the result of long‐term accumulation of species with low extinction rate (the “museum hypothesis”) or a consequence of accelerated diversification, compared to the lowlands (the “cradle hypothesis”) (Schwery et al. 2015). However, species richness patterns alone are insufficient to distinguish between these alternative diversification scenarios. It was worth noting that our analyses support alpine origin of the largest genus *Calanthe*, a cosmopolitan genus. Several early‐diverging lineages are confined to high‐elevation habitats, including *C. delavayi* (2700–3450 m), and basal clade of the Clade III–C ( *C. arcuata* , and *C. taibainensis*) and Clade III–D (*C. alpine*, and *C. metoensis*; Figure 5B). The persistence of these ancient lineages in montane environments suggests long‐term survival and reduced extinction, consistent with a mountain “museum” scenario. In contrast, other montane clades, particularly Clade III–D, are characterized by extremely short internodes and recent divergence times, indicating rapid speciation. This pattern is consistent with a “cradle” role of montane regions, where ecological heterogeneity and Quaternary climatic fluctuations may have promoted accelerated lineage diversification.

Mountainous speciation models have been tested on many organisms, like Afrotropical and Neotropical avifaunas, *Ithomiola* butterflies, Ericaceae, *Dendrobium* (Fjeldså 1994; Hall 2005; Schwery et al. 2015; Xiang et al. 2016); they all show the young lineage diversifying in high land area and old basal species existing in lowland, while *Calanthe* is the complete opposite to this observation in terms of speciation. Previous studies showed that the invasion of high altitude places by plants is one of the diversification impetuses of Orchidaceae (Givnish et al. 2016). The peak of *Calanthe* species richness at mid‐elevations may therefore reflect the combined outcome of long‐term lineage persistence and recent diversification, rather than serving as direct evidence for either process alone.

There were two kinds of diversification in the *Calanthe*: “long branch clade (LBC)” (Clade III–C) and “short branch clade (SBC)” (Clade III–D). During the mid‐Miocene, numerous speciation events and species accumulation leading to extant taxa that may be observed within the LBC, whereas for the SBC there is a period of apparent stasis. Combined with its altitude‐related adaptation, we found an evolutionary direction of LBC to push for adaptability to wider altitude ranges and lower altitude. Plants respond to climate change either by dispersing/changing geographical distribution, adapting and evolving, creating new species, or facing extinction (Jump and Peñuelas 2005). The *Calanthe* diverged at the end of the mid‐Miocene at optimum climate conditions (ca. 14.8 Ma) (Tolley et al. 2008), during the rapid upward movement of the Qinghai‐Tibet Plateau and during the subsequent and rapid climate decline (Wen et al. 2014; Zachos et al. 2001). We postulated that the LBC speciation arose by the selective pressure of the climate and the new species were distributed at low altitudes because of the adaptation to the colder climate. Mountainous areas contain high ecological heterogeneity and might have been better capable of adjusting to varying climatic conditions through altitude range fluctuations over short geographical distances than low altitude land (Russell et al. 2010). Diversification in LBC gradual species accumulation through time while SBC from rapid radiation, investigating the different diversification process between two clades in the same genus would be of great evolutionary interest.

## Author Contributions

**Jun‐Wen Zhai:** formal analysis (equal), investigation (equal), visualization (equal), writing – original draft (equal), writing – review and editing (equal). **Yanqiong Chen:** formal analysis (equal), investigation (equal), visualization (equal), writing – original draft (equal), writing – review and editing (equal). **Qiang Wang:** investigation (equal), resources (equal). **Jiahao Zheng:** investigation (equal), resources (equal). **Chen Chen:** investigation (equal), resources (equal). **Pingting Guo:** investigation (equal), resources (equal). **Hongyu Ji:** investigation (equal), resources (equal). **Jianglin Miao:** investigation (equal), resources (equal). **Muyang Li:** investigation (equal), resources (equal). **Minghe Li:** writing – review and editing (equal). **Shasha Wu:** writing – review and editing (equal). **Siren Lan:** conceptualization (equal), project administration (equal), supervision (equal), writing – review and editing (equal). **Zhong‐Jian Liu:** conceptualization (equal), project administration (equal), supervision (equal), writing – review and editing (equal).

## Funding

This work was supported by the construction and management of the research center for the protection and utilization of orchids in Motuo County, Xizang Autonomous Region, China (KH230350A), the Project of Biodiversity Survey and Assessment of the Collabium (OU202203).

## Ethics Statement

The authors have nothing to report.

## Consent

The authors have nothing to report.

## Conflicts of Interest

The authors declare no conflicts of interest.

## Supporting information


**Figure S1:** Bayesian inference based on the nrITS. Numbers near the nodes are Bayesian posterior probabilities and bootstrap percentages (PP left, BS_ML_ middle, and BS_MP_ right). An asterisk (*) indicates that the node has 1.0 posterior probability or 100%.
**Figure S2:** Bayesian inference based on the single‐copy SNPs. Numbers near the nodes are Bayesian posterior probabilities and bootstrap percentages (PP left, BS_ML_ middle, and BS_MP_ right). An asterisk (*) indicates that the node has 1.0 posterior probability or 100%.
**Table S1:** Characteristics of the *Calanthe* alliance plastome generated in this study. The accession number of plastome and nrITS. The bold indicate the new data obtained in this study.
**Table S2:** State of the *Calanthe* alliance used in this study.
**Table S3:** Morphological characteristics of the *Calanthe* alliance used in this study.
**Table S4:** Divergence times (Mya) of the mainly groups in *Calanthe* alliance, with result of ancestral reconstruction using the S‐DIVA and DEC.

## Data Availability

All data supporting the findings of this study are available within the article and in the Supporting Information. The accession number of plastome and nrITS in this study can be found in Table S1. The code used in study is archived on Zenodo and accessible at the following: https://doi.org/10.5281/zenodo.17152960.
