# Supplementary material for: Phylogeny and Biogeography of Calanthe Shed New Light on Alpine Origin and Radiation History of Calanthe Alliance
Source: Ecol Evol. 2026 Mar 18;16(3):e73301. doi: 10.1002/ece3.73301 (PMC13093556; doi:10.1002/ece3.73301)
Supplement: Supplementary file 1 — Figure S1: Bayesian inference based on the nrITS. Numbers near the nodes are Bayesian posterior probabilities and bootstrap percentages (PP left, BSML middle, and BSMP right). An asterisk (*) indicates that the node has 1.0 posterior probability or 100%. Figure S2: Bayesian inference based on the single‐copy SNPs. Numbers near the nodes are Bayesian posterior probabilities and bootstrap percentages (PP left, BSML middle, and BSMP right). An asterisk (*) indicates that the node has 1.0 posterior probability or 100%. Table S1: Characteristics of the Calanthe alliance plastome generated in this study. The accession number of plastome and nrITS. The bold indicate the new data obtained in this study. Table S2: State of the Calanthe alliance used in this study. Table S3: Morphological characteristics of the Calanthe alliance used in this study. Table S4: Divergence times (Mya) of the mainly groups in Calanthe alliance, with result of ancestral reconstruction using the S‐DIVA and DEC. [file ECE3-16-e73301-s001.zip › Supplements.docx]

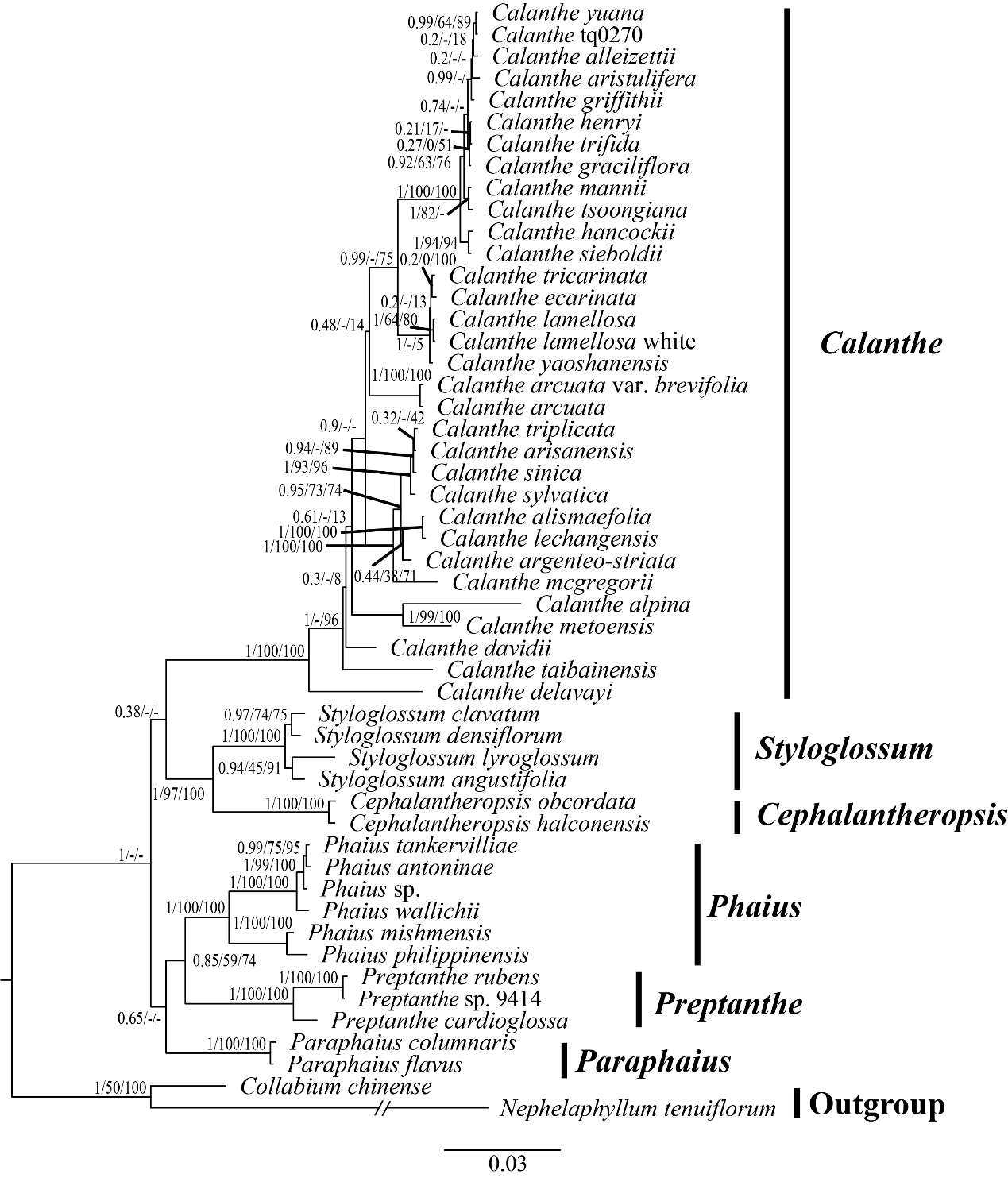


**Figure S1** Bayesian inference based on the nrITS. Numbers near the nodes are Bayesian posterior probabilities and bootstrap percentages (PP left, BS_ML_ middle, and BS_MP_ right). An asterisk (*) indicates that the node has 1.0 posterior probability or 100%


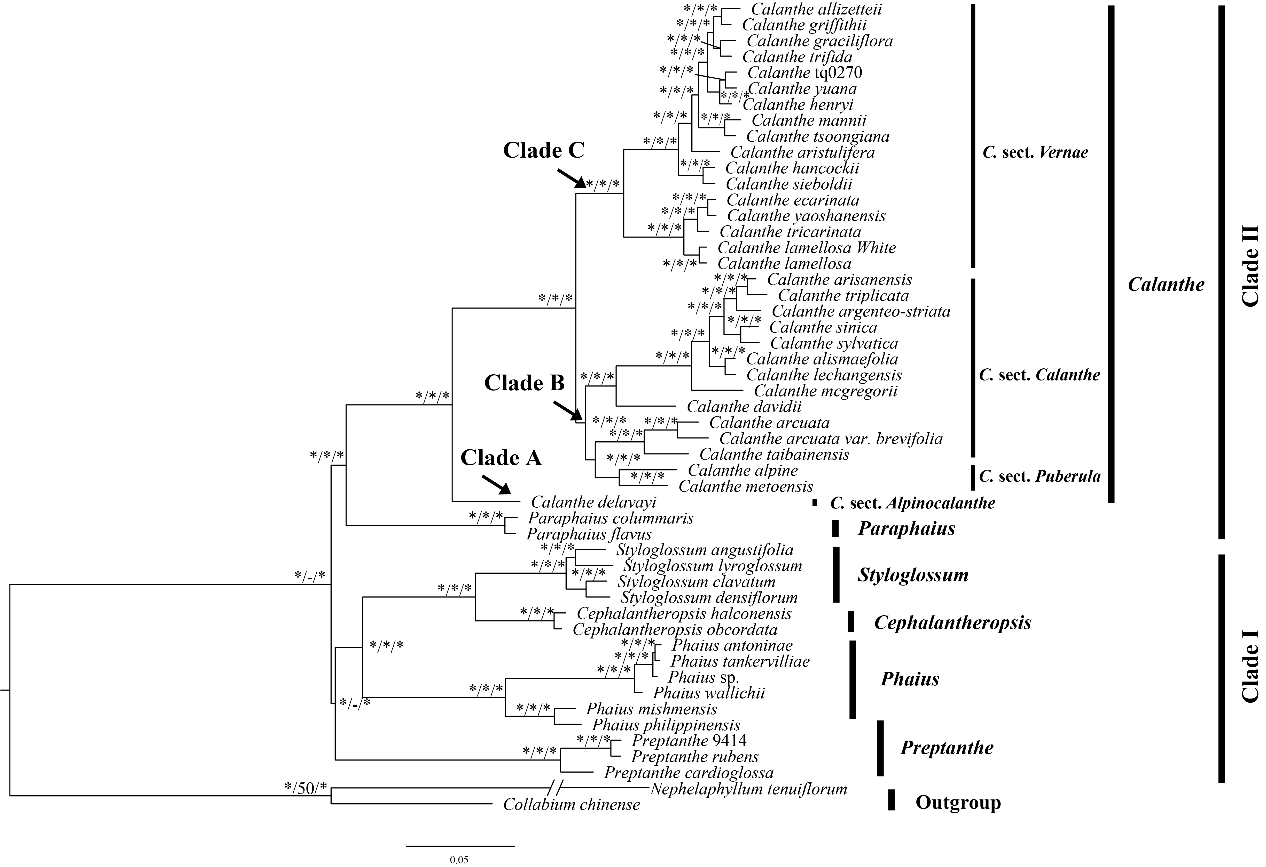


**Figure S2** Bayesian inference based on the single-copy SNPs. Numbers near the nodes are Bayesian posterior probabilities and bootstrap percentages (PP left, BS_ML_ middle, and BS_MP_ right). An asterisk (*) indicates that the node has 1.0 posterior probability or 100%.

**Table S1** Characteristics of the *Calanthe* alliance plastome generated in this study. The accession number of plastome and nrITS. The bold indicate the new data obtained in this study.

| Organism | Location | Sequence voucher | Genome size/bp | LSC length  /bp | IR length  /bp | SSC length  /bp | GC content  /% | Accession number | |
| --- | --- | --- | --- | --- | --- | --- | --- | --- | --- |
|  |  |  |  |  |  |  |  | Plastome | nrITS |
| *Calanthe alismaefolia* Lindl. | Yunnan, China | J. W. Zhai 056 (IBSC) | 158,078 | 87,097 | 26,228 | 18,525 | 36.70% | **OK180385** | **OK183549** |
| *Calanthe alleizettii* Gagnep. | Vietnam | Y.Q.Chen 131(FAFU) | 158,251 | 87,125 | 26,359 | 18,408 | 36.70% | **OK180386** | **OK183583** |
| *Calanthe alpina* Hook.f. ex Lindl. | Sichuan, China | Y.Q.Chen 004 (FAFU) | 153,974 | 83,671 | 26,273 | 17,757 | 36.70% | **OK180387** | **OK183572** |
| *Calanthe arcuata* Rolfe | Shanxi,China | Y.Q.Chen 006 (FAFU) | 158,735 | 87,398 | 26,429 | 18,479 | 36.60% | **OK180389** | **OK183582** |
| *Calanthe arcuata* var. *brevifolia* Z. H. Tsi | Sichuan, China | Y.Q.Chen 007 (FAFU) | 158,695 | 87,595 | 26,324 | 18,452 | 36.70% | **OK180390** | **OK183576** |
| *Calanthe argenteo-striata* C. Z. Tang et S. J. Cheng | Yunnan, China | J. W. Zhai 008 (IBSC) | 158,139 | 86,752 | 26,436 | 18,515 | 36.80% | **OK180391** | **OK183544** |
| *Calanthe arisanensis* Hayata. | Taiwan | J. W. Zhai 175 (NOCC) | 158,876 | 87,495 | 26,373 | 18,635 | 36.70% | **OK180392** | **OK183569** |
| *Calanthe aristulifera* Rchb.f. | Yunnan, China | Y.Q.Chen 072 (FAFU) | 158,300 | 87,369 | 26,251 | 18,429 | 36.70% | **OK180393** | **OK183557** |
| *Calanthe davidii* Franch. | Yunnan, China | J. W. Zhai 063 (IBSC) | 159,014 | 87,857 | 26,284 | 18,589 | 36.60% | MN708353 | **OK183548** |
| *Calanthe delavayi* Finet. | NOCC | Z. J. Liu 5323 (NOCC) | 150,181 | 83,411 | 25,216 | 16,338 | 36.90% | MK388860 | **OK183573** |
| *Calanthe ecarinata* Rolfe ex Hemsl. | Sichuan, China | Y.Q.Chen 017 (FAFU) | 158,452 | 87,200 | 26,348 | 18,556 | 36.70% | **OK180397** | **OK183581** |
| *Calanthe graciliflora* Hayata | Guangdong, China | J. W. Zhai 045 (IBSC) | 158,327 | 87,397 | 26,251 | 18,428 | 36.70% | **OK180398** | **OK183551** |
| *Calanthe griffithii* Lindl. | Yunnan, China | Y.Q.Chen 104 (FAFU) | 158,274 | 87,090 | 26,383 | 18,418 | 36.70% | **OK180399** | **OK183575** |
| *Calanthe hancockii* Rolfe | Yunnan, China | J. W. Zhai 077 (IBSC) | 158,258 | 87,127 | 26,363 | 18,405 | 36.70% | **OK180400** | **OK183550** |
| *Calanthe henryi* Rolfe | Wuhan, China | Y.Q.Chen 108 (FAFU) | 158,237 | 87,125 | 26,345 | 18,422 | 36.70% | **OK180401** | **OK183562** |
| *Calanthe lamellosa* Rolfe | Shanxi, China | Y.Q.Chen10502 (FAFU) | 158,200 | 86,887 | 26,473 | 18,367 | 36.70% | **OK180402** | **OK183584** |
| *Calanthe lamellosa* Rolfe whiteflower | Shanxi, China | Y.Q.Chen 132 (FAFU) | 158,494 | 87,395 | 26,261 | 18,577 | 36.70% | **OK180403** | **OK183587** |
| *Calanthe lechangensis* Z.H.Tsi & Tang | Guangdong, China | J. W. Zhai 002 (IBSC) | 158,222 | 87,449 | 26,119 | 18,535 | 36.70% | **OK180404** | **OK183556** |
| *Calanthe mannii* Hook.f. | Yunnan, China | Z. J. Liu 4296 (NOCC) | 158,062 | 87,143 | 26,252 | 18,415 | 36.70% | **OK180405** | **OK183558** |
| *Calanthe mcgregorii* Ames | Philippinen | Y.Q.Chen 048 (FAFU) | 158,891 | 87,428 | 26,405 | 18,653 | 36.70% | **OK180406** | **OK183579** |
| *Calanthe metoensi* Z.H.Tsi & K.Y.Lang | Yunnan, China | Y.Q.Chen 122 (FAFU) | 158,553 | 87,424 | 26,445 | 18,239 | 36.60% | **OK180407** | **OK183591** |
| *Calanthe sieboldii* Decne. ex Regel | Hunan, China | J. W. Zhai 168 (NOCC) | 158,299 | 87,180 | 26,345 | 18,429 | 36.70% | **OK180408** | **OK183564** |
| *Calanthe sinica* Z.H.Tsi | Yunnan, China | J. W. Zhai 075 (IBSC) | 158,843 | 87,518 | 26,387 | 18,551 | 36.70% | **OK180409** | **OK183545** |
| *Calanthe* sp. tq0270 | Sichuan, China | Y.Q.Chen 089 (FAFU) | 158,238 | 87,126 | 26,345 | 18,422 | 36.70% | **OK180384** | **OK183580** |
| *Calanthe sylvatica* (Thouars) Lindl. | Guangxi, China | J. W. Zhai 062 (IBSC) | 158,845 | 87,310 | 26,492 | 18,551 | 36.70% | **OK180410** | **OK183546** |
| *Calanthe taibaishanensis* M. Guo, J.W. Zhai & L.J. Chen | Shanxi,China | Y.Q.Chen 111 (FAFU) | 158,172 | 87,438 | 26,203 | 18,328 | 36.60% | **OK180411** | **OK183586** |
| *Calanthe tricarinata* Lindl. | Guizhou, China | J. W. Zhai 019 (IBSC) | 158,354 | 87,292 | 26,268 | 18,526 | 36.70% | **OK180412** | **OK183563** |
| *Calanthe trifida* Tang & F.T.Wang | Fujian, China | Y.Q.Chen 123 (FAFU) | 158,330 | 87,218 | 26,345 | 18,422 | 36.70% | **OK180413** | **OK183592** |
| *Calanthe triplicata* (Willemet) Ames | Hainan, China | J. W. Zhai 013 (IBSC) | 158,853 | 87,317 | 26,492 | 18,552 | 36.70% | KF753635 | **OK183555** |
| *Calanthe tsoongiana* Tang & F.T.Wang | Zhejiang, China | J. W. Zhai 044 (IBSC) | 158,042 | 87,160 | 26,234 | 18,414 | 36.70% | **OK180414** | **OK183565** |
| *Calanthe yuana* Tang & F.T.Wang | Sichuan, China | Y.Q.Chen 045 (FAFU) | 158,255 | 87,080 | 26,383 | 18,409 | 36.70% | **OK180416** | **OK183574** |
| *Cephalantheropsis halconensis* (Ames) S.S.Ying | Taiwan | J. W. Zhai 176 (NOCC) | 157,880 | 86,758 | 26,279 | 18,564 | 36.80% | **OK180417** | **OK183570** |
| *Cephalantheropsis obcordata* (Lindl.) Ormerod | Yunnan, China | J. W. Zhai 017 (IBSC) | 157,919 | 86,651 | 26,424 | 18,420 | 36.80% | MN708351 | **OK183554** |
| *Parahaius flavus* (Blume) Lindl. | NOCC | Z. J. Liu 3852 (NOCC) | 158,561 | 87,435 | 26,261 | 18,604 | 36.80% | **OK180422** | **OK183560** |
| *Paraphaius columnaris* C.Z.Tang & S.J.Cheng | Yunnan, China | J. W. Zhai 016 (IBSC) | 157,982 | 87,023 | 26,379 | 18,201 | 36.90% | **OK180421** | **OK183553** |
| *Phaius* sp. | Taiwan | J. W. Zhai 174 (NOCC) | 158,296 | 86,915 | 26,512 | 18,357 | 37.00% | **OK180425** | **OK183568** |
| *Phaius antoninae* P.Balzer | Philippinen | Y.Q.Chen 065 (FAFU) | 158,235 | 86,621 | 26,618 | 18,378 | 37.00% | **OK180420** | **OK183578** |
| *Phaius mishmensis* (Lindl. & Paxton) Rchb.f. | NOCC | Z. J. Liu 3234 (NOCC) | 158,382 | 86,736 | 26,603 | 18,440 | 36.90% | **OK180423** | **OK183559** |
| *Phaius philippinensis* N.E.Br. | Philippinen | Y.Q.Chen 068 (FAFU) | 157,979 | 86,064 | 26,708 | 18,499 | 37.00% | **OK180424** | **OK183577** |
| *Phaius tankervilleae* (Banks) Blume | NOCC | Z. J. Liu 3417 (NOCC) | 158,229 | 86,638 | 26,617 | 18,357 | 37.00% | MN708349 | **OK183561** |
| *Phaius wallichii* Lindl. | NOCC | Z. J. Liu 5321 (NOCC) | 158,234 | 86,576 | 26,627 | 18,404 | 37.00% | **OK180426** | **OK183571** |
| *Preptanthe cardioglossa* (Schltr.) T.Yukawa & P.J.Cribb | Vietnam | J. W. Zhai 167 (IBSC) | 159,299 | 87,868 | 26,500 | 18,431 | 36.70% | **OK180394** | **OK183588** |
| *Preptanthe rubens* (Ridl.) Ridl. | Vietnam | J. W. Zhai 014 (IBSC) | 158,215 | 87,602 | 26,055 | 18,503 | 36.70% | MN708352 | **OK183552** |
| *Preptanthe* sp. 9414 | Vietnam | Z. J. Liu 9414(NOCC) | 158,211 | 87,316 | 26,153 | 18,589 | 36.70% | **OK180383** | **OK183589** |
| *Styloglossum angustifolia* (Blume) T.Yukawa & P.J.Cribb | Taiwan | J. W. Zhai 179 (NOCC) | 157,126 | 86,598 | 26,117 | 18,294 | 36.80% | **OK180388** | **OK183590** |
| *Styloglossum clavatum* (Lindl.) T.Yukawa & P.J.Cribb, | Guangdong, China | J. W. Zhai 146 (IBSC) | 157,641 | 87,004 | 26,145 | 18,347 | 36.80% | **OK180395** | **OK183547** |
| *Styloglossum densiflorum* (Lindl.) T.Yukawa & P.J.Cribb | Taiwan | J. W. Zhai 173 (NOCC) | 157,609 | 86,896 | 26,182 | 18,349 | 36.80% | **OK180396** | **OK183567** |
| *Styloglossum lyroglossum* (Rchb.f.) T.Yukawa & P.J.Cribb | Hainan, China | J. W. Zhai 144 (IBSC) | 156,036 | 85,526 | 26,128 | 18,254 | 36.90% | MN708350 | **OK183566** |
| *Collabium chinense* (Rolfe) Tang & F.T.Wang | NOCC | Z. J. Liu 4823 (NOCC) | 157,758 | 86,299 | 26,452 | 18,555 | 37.20% | **OK180418** | **OK183543** |
| *Nephelaphyllum tenuiflorum* Blume | NOCC | Z. J. Liu 4009 (NOCC) | 151,779 | 84,420 | 25,988 | 15,383 | 37.20% | **OK180419** | **OK183593** |

**Table S2** State of the *Calanthe* alliance used in this study.

| Name | State | Elevation | Distribution |
| --- | --- | --- | --- |
| Calanthe alismaefolia | AB | 800-1700m | India (North-west Himalayas, Sikkim), Bhutan, China (SE Xizang (Tibet), Sichuan, Hubei, Hunan, NW to SE Yunnan, Taiwan), Japan, Vietnam. |
| *Calanthe alleizettii* | AB | 1000-1700m | Yunnan province of China and Vietnam |
| *Calanthe alpina* | AB | 1500-4000m | Nepal, India (Himalayas, Nagaland, Sikkim), China (Hupei, S Shaanxi, S Gansu, Sichuan, S-SE Xizang, NW to SW Yunnan, Taiwan), Japan (C, S). |
| *Calanthe arcuata* | A | 1400-3100m | China (S Gansu, Guizhou, W Hupei, Hunan, S Shaanxi, W Sichuan, W to NW Yunnan, E Xizang (Tibet), Taiwan). |
| *Calanthe arcuata* | A | 1500-1700m | China (S Gansu, Guizhou, W Hupei, Hunan, S Shaanxi, W Sichuan. |
| *Calanthe argenteo-striata* | AB | 500-1200m | China (Guangdong, Guangxi, Guizhou, SE Yunnan), Vietnam. |
| *Calanthe arisanensis* | A | 1500-4000m | Nepal, India (Himalayas, Nagaland, Sikkim), China (Hupei, S Shaanxi, S Gansu, Sichuan, S-SE Xizang, NW to SW Yunnan, Taiwan), Japan (C, S). |
| *Calanthe aristulifera* | A | 1400-3100m | China (S Gansu, Guizhou, W Hupei, Hunan, S Shaanxi, W Sichuan, W to NW Yunnan, E Xizang (Tibet), Taiwan). |
| *Calanthe davidii* | AB | 500-3300m | Nepal, India (W. Himalayas), China (S Gansu, Guizhou, Hubei, NW Hunan, S Shaanxi, Sichuan, SE Xizang (Tibet), W to SE Yunnan, Taiwan), Japan (S). |
| *Calanthe delavayi* | A | 2700-3450m | China (Yunnan). |
| *Calanthe ecarinata* | A | 1675-2500m | China (W Sichuan). |
| *Calanthe graciliflora* | A | 600-2400m | China (Anhui, N Fujian, Guangdong, Guangxi, Guizhou, Hong Kong, Hubei, Hunan, Jiangxi, SW Sichuan, SW to SE Yunnan, Zhejiang, Taiwan). |
| *Calanthe griffithii* | AB | 1500-3000m | Nepal, Bhutan, SW China (Yunnan, SE Xizang (Tibet)), India (N Punjab, Kashmir, Sikkim, Nagaland), Myanmar. |
| *Calanthe hancockii* | AB | 1000-3600m | China, (N Guangxi, SW Sichuan, NW to SE Yunnan), Myanmar. |
| *Calanthe henryi* | A | 1600-2100m | Sichuan and Hubei |
| *Calanthe lamellosa* | A | 1800 to 2300 | north Guanxi, Hubei, Sichuan and western to southeastern Yunnan |
| *Calanthe lamellosa whiteflower* | A | 1800 to 2300 | north Guanxi, Hubei, Sichuan and western to southeastern Yunnan |
| *Calanthe lechangensis* | A | 100m | China (N Guangdong). |
| *Calanthe mannii* | AB | 2000-2400m | Nepal, Bhutan, India (Sikkim, Khasia Hills, Nagaland, Manipur and other parts of the Himalayas), China (E Guangdong, NE Guangxi, SW Guizhou, NE Yunnan, SW Hubei, N Jiangxi, Sichuan, SE to S Xizang (Tibet)), Myanmar, Vietnam, Japan. |
| *Calanthe mcgregorii* | C | 330m | Philippines. |
| *Calanthe metoensi* | A | 2200 to 2300 | Tibet and Yunnan |
| *Calanthe sieboldii* | A | 0-1500m | China (Hunan, Taiwan (N & SW)), Japan (S. Honshu, Shikoku, Kyushsu, Ryukyu Islands), Korea (SC, S). |
| *Calanthe sinica* | A | 1050-1100m | SE Yunnan (Wenshan). |
| *Calanthe sp.* |  |  |  |
| *Calanthe sylvatica* | ABCD | 800-2000m | Tropical West Africa (Annobon Is., Fernando Po Is., Sao Tomé Is., Angola, Burundi, Cameroon (W & E), Equatorial Guinea, Gabon, Guinea, Nigeria, D.R. Congo, Zaire, Tropical East Africa (Kenya, Malawi, Rwanda, Tanzania, Uganda, Zambia, Zimbabwe), Swaziland, South Africa (Cape Province, Eastern Cape, Natal, Transvaal), West Indian Ocean Islands (Comoro Islands, Madagascar, Mauritius, Réunion,Seychelles). |
| *Calanthe taibaishanensis* | A |  |  |
| *Calanthe tricarinata* | AB | 300-3500m | Nepal, Bhutan, Pakistan (Kashmir), India (Arunachal Pradesh, Nagaland, Meghalaya, Manipur, Mizoram, Sikkim), N. Myanmar, China (S Gansu, Hubei, S Shaanxi, Guizhou, Sichuan, SE Xizang (Tibet)), Yunnan), Taiwan, Thailand, Japan (including Ryuku Islands), Korea. |
| *Calanthe trifida* | AB | 1700 m | Yunnan province of China and Myanmar |
| *Calanthe triplicata* | ABC | 0-3000m | Bhutan, India (Assam), Myanmar (Maymyo Plateau), China (S Fujian, SW & N Guangdong, Guangxi, Hainan, Hong Kong, Taiwan, N to SE Yunnan), Japan (Ryukyu Islands, Okinawa), Cambodia, Laos, Thailand (widely distributed), Vietnam, Borneo (Sabah, Sarawak), Peninsular Malaysia, Singapore, Java, Sumatra, Maluku, Sulawesi, Papua New Guinea, Philippines (Luzon, Mindanao, Panoy, Sibutu), Solomon Islands (Choiseul, Malaita), Vanuatu (Aeityum, Banks Is., Efate Is., Espiritu Santo, Pentcost, Tanna), Fiji (Vanua Levu, Viti Levu), Guam, Samoa (Savi’i, Upulu), New Caledonia, Australia (New South Wales, Queensland)),. |
| *Calanthe tsoongiana* | A | 400-1500m | China (N Fujian, Jiangxi, Zhejiang). |
| *Calanthe yuana* | A | 1800m | China (W Hubei, Sichuan). |
| *Cephalantheropsis halconensis* | AC | 1250m | China (S & W Guanxi, Yunnan and S & E Xizang, Taiwan), Philippines. |
| *Cephalantheropsis obcordata* | ABC | 1300-1450m | China (Fujian, Guangdong, Hainan and Yunnan,Taiwan), Japan, Bangladesh, Laos, Myanmar, Thailand, Vietnam, Hong Kong, Malaysia, the Moluccas and the Philippines. |
| *Parahaius flavus* | ABC | 300-2500m | India eastward thruogh most of S & E Asia to New Guinea, north to Taiwan. |
| *Paraphaius columnaris* | A | 230-1700m | China (S & W Guizhou, N Guangdong, S Yunnan). |
| *Phaius sp.* |  |  |  |
| *Phaius antoninae* | C | 600m | Philippines. |
| *Phaius mishmensis* | ABC | 500-2000m | China, Assam India, eastern Himalayas, Myanamar, Thailand, Laos, Vietnam, Philippines, Taiwan and the Ryukyus. |
| *Phaius philippinensis* | C | ~1300m | Philippines. |
| *Phaius tankervilleae* | ABC | 700-1800m | China (Fujian, Guangdong, Guangxi, Hainan, Xizang and Yunnan, Hong Kong, Taiwan), the Pacific Islands, Malaysia and Indonesia. |
| *Phaius wallichii* | A | 750-1000m | Northern India, Nepal, Bhutan, Indo-China and southern China. |
| *Phaius takeoi* | AB | 500-1400M | Myanmar, Thailand, south Yunnan, Vietnam and Taiwan |
| *Preptanthe cardioglossa* | BC | 400-1580m | Thailand, Vietnam (Dalat), Laos (Phu Set, Saravane). |
| *Preptanthe rubens* | BC | 0-1800m | Thailand (Central & Peninsular), Vietnam, Peninsular Malaysia (Langkawi Islands, Perlis), Borneo, Philippines (Luzon, Palawan). |
| *Preptanthe* sp. |  |  |  |
| *Styloglossum angustifolia* | ABC | 1000-2000m | China (Guangdong, Hainan, Taiwan (S)), Java, Sumatra, Peninsular Malaysia (Kedah, Pahang, Perak), Vietnam, Philippines (Luzon, Mindoro). |
| *Styloglossum clavatum* | AB | 460-1660m | India (Khasia Hills, Sikkim, Darjeeling), Bangladesh, China (Fujian, Guangdong, Hainan, Guangxi, S Yunnan, SE Xizang), Myanmar, Peninsular Malaysia (Cameron Highlands), Thailand (Doi Sutep, Pangan), Vietnam (Nam Dao). |
| *Styloglossum densiflorum* | AB | 1000-3050m | Nepal, Bhutan, India (Arunachal Pradesh, Meghalaya, Nagaland, Sikkim), Bangladesh, Myanmar, China (Guangdong, Guangxi, Hainan, Sichuan, S Xizang, Yunnan, Taiwan), Japan (Ryukyu), Thailand, Vietnam. |
| *Styloglossum lyroglossum* | ABC | 1000-1770m | China (Hainan, Taiwan), Okinawa, NE India, Upper Myanmar, Thailand, Laos, Borneo (Sabah), Cambodia, Vietnam, Peninsular Malaysia, Philippines (Negros). |
| *Collabium chinense* | AB | 600-1000m | China (S Fujian, S Guangdong, S Guangxi, Hainan, SE Xizang, and SE Yunnan, Hong Kong, Taiwan), Laos. |
| *Nephelaphyllum tenuiflorum* | A | 900m | Thailand, Malaysia, Borneo, Java, Sumatra and Vietnam |

**Table S3** Morphological characteristics of the *Calanthe* alliance used in this study.

| Organism | Lip | | | spur |
| --- | --- | --- | --- | --- |
|  | adnate to column base | lobe | appendage |  |
| *Calanthe alismaefolia* Lindl. | 0 | 2 | 1 | 1 |
| *Calanthe alleizettii* Gagnep. | 0 | 2 | 1 | 3 |
| *Calanthe alpina* Hook.f. ex Lindl. | 0 | 0 | 0 | 3 |
| *Calanthe arcuata* Rolfe | 0 | 1 | 1 | 1 |
| *Calanthe arcuata* var*. brevifolia* Z. H. Tsi | 0 | 1 | 1 | 1 |
| *Calanthe argenteo-striata* C. Z. Tang et S. J. Cheng | 0 | 2 | 1 | 2 |
| *Calanthe arisanensis* Hayata*.* | 0 | 1 | 1 | 2 |
| *Calanthe aristulifera* Rchb.f. | 0 | 1 | 1 | 3 |
| *Calanthe davidii* Franch*.* | 0 | 2 | 1 | 2 |
| *Calanthe delavayi* Finet*.* | 0 | 0 | 1 | 2 |
| *Calanthe ecarinata* Rolfe ex Hemsl. | 0 | 1 | 0 | 0 |
| *Calanthe graciliflora* Hayata | 0 | 1 | 1 | 2 |
| *Calanthe griffithii* Lindl*.* | 0 | 1 | 1 | 1 |
| *Calanthe hancockii* Rolfe | 0 | 1 | 1 | 1 |
| *Calanthe henryi* Rolfe | 0 | 1 | 1 | 2 |
| *Calanthe lamellosa* Rolfe | 0 | 1 | 1 | 1 |
| *Calanthe lamellosa* Rolfe whiteflower | 0 | 1 | 1 | 1 |
| *Calanthe lechangensis* Z.H.Tsi & Tang | 0 | 1 | 1 | 1 |
| *Calanthe mannii* Hook.f. | 0 | 1 | 1 | 1 |
| *Calanthe mcgregorii* Ames | 0 | 2 | 1 | 1 |
| *Calanthe metoensi* Z.H.Tsi & K.Y.Lang | 0 | 1 | 1 | 2 |
| *Calanthe sieboldii* Decne. ex Regel | 0 | 1 | 1 | 2 |
| *Calanthe sinica* Z.H.Tsi | 0 | 1 | 1 | 3 |
| *Calanthe* sp. tq0270 | 0 | 1 | 1 | 2 |
| *Calanthe sylvatica* (Thouars) Lindl. | 0 | 2 | 1 | 3 |
| *Calanthe taibaishanensis* M. Guo, J.W. Zhai & L.J. Chen | 0 | 1 | 1 | 2 |
| *Calanthe tricarinata* Lindl. | 0 | 1 | 1 | 0 |
| *Calanthe trifida* Tang & F.T.Wang | 0 | 1 | 1 | 1 |
| *Calanthe triplicata* (Willemet) Ames | 0 | 2 | 1 | 2 |
| *Calanthe tsoongiana* Tang & F.T.Wang | 0 | 1 | 0 | 0 |
| *Calanthe yuana* Tang & F.T.Wang | 0 | 1 | 0 | 1 |
| *Cephalantheropsis halconensis* (Ames) S.S.Ying | 1 | 1 | 1 | 0 |
| *Cephalantheropsis obcordata* (Lindl.) Ormerod | 1 | 1 | 1 | 0 |
| *Collabium chinense* (Rolfe) Tang & F.T.Wang | 1 | 1 | 1 | 1 |
| *Nephelaphyllum tenuiflorum* Blume | 1 | 1 | 1 | 1 |
| *Parahaius flavus* (Blume) Lindl. | 1 | 0 | 1 | 1 |
| *Paraphaius columnaris* C.Z.Tang & S.J.Cheng | 1 | 1 | 1 | 1 |
| *Phaius antoninae* P.Balzer | 1 | 0 | 1 | 1 |
| *Phaius mishmensis* (Lindl. & Paxton) Rchb.f. | 1 | 1 | 1 | 2 |
| *Phaius philippinensis* N.E.Br. | 1 | 0 | 1 | 1 |
| *Phaius* sp. | 1 | 1 | 1 | 1 |
| *Phaius tankervilleae* (Banks) Blume | 1 | 1 | 1 | 1 |
| *Phaius wallichii* Lindl*.* | 1 | 1 | 1 | 2 |
| *Preptanthe cardioglossa* (Schltr.) T.Yukawa & P.J.Cribb | 0 | 1 | 1 | 3 |
| *Preptanthe rubens* (Ridl.) Ridl. | 0 | 2 | 0 | 3 |
| *Preptanthe* sp. 9414 | 0 | 1 | 1 | 3 |
| *Styloglossum angustifolia* (Blume) T.Yukawa & P.J.Cribb | 0 | 2 | 1 | 1 |
| *Styloglossum clavatum* (Lindl.) T.Yukawa & P.J.Cribb, | 0 | 1 | 1 | 2 |
| *Styloglossum densiflorum* (Lindl.) T.Yukawa & P.J.Cribb | 0 | 1 | 1 | 2 |
| *Styloglossum lyroglossum* (Rchb.f.) T.Yukawa & P.J.Cribb | 0 | 1 | 1 | 1 |

Lip adnate to column base: exsit=0, absent = 1;

Lip lobe: lip unlobe = 0, lip lobed with mid-lobe unlobed = 1, lip lobed with mid-lobe lobed = 2;

Lip appendage: absent = 0; exsit = 1;

Spur: absent = 0; short length (≤ 10 mm) =1; middle length (＜20 mm, ＞10mm) = 2, long length (≥ 20 mm) = 3.

**Table S4.** Divergence times (Mya) of the mainly groups in *Calanthe* alliance, with result of ancestral reconstruction using the S-DIVA and DEC.

| Node | Age estimates Median(95%HPD) | | Ancestral area | |
| --- | --- | --- | --- | --- |
|  | Stem | Crown | S-DIVA | DEC |
| *Preptanthe* | 21.22(16.52-29.11) | 6,82(2.39-11.92) | A(0.5), B(0.5) | AB(0.54), B(0.46) |
| *Cephalantheropsis* | 11.08(7.79-14.66) | 0.59(0.28-1.04) | A(1.0) | AC(0.79), A(0.21) |
| *Styloglossum* | 11.08(7.79-14.66) | 4.17(2.02-6.40) | A(1.0) | A(0.68), AB(0.22), AC(0.10) |
| *Phaius* | 16.74(12.98-21.11) | 7.43(0.70-10.39) | AC(0.5), A(0.5) | A(0.48), AC(0.37), C(0.15) |
| *Paraphaius* | 17.41(13.59-21.93) | 1.27(0.51-2.81) | A(1.0) | A(1.0) |
| *Calanthe* | 19.42(15.11-24.20) | 15.49(11.95-19.62) | A(1.0) | A(1.0) |
